# Factors associated with satisfaction at work in Psychosocial Care Centers^1^

**DOI:** 10.1590/0104-1169.3474.2500

**Published:** 2014

**Authors:** Sonia Regina da Costa Lapischies, Vanda Maria da Rosa Jardim, Luciane Prado Kantorski

**Affiliations:** 2 MSc, RN, Secretaria Municipal de Saúde de Pelotas, Pelotas, RS, Brazil; 3 PhD, Adjunct Professor, Faculdade de Enfermagem, Universidade Federal de Pelotas, Pelotas, RS, Brazil; 4 PhD, Associate Professor, Faculdade de Enfermagem, Universidade Federal de Pelotas, Pelotas, RS, Brazil

**Keywords:** Job Satisfaction, Mental Health, Community Mental Health Services, Work

## Abstract

**OBJECTIVES:**

to analyze the prevalence of satisfaction at work and identify associated factors in Psychosocial Care Centers.

**METHOD:**

cross-sectional study involving 546 workers from 40 Psychosocial Care Centers in the South of Brazil. The satisfaction was identified based on the Assessment Scale of Satisfaction in the Mental Health Team and a logistic regression model was used for the adjusted data analysis.

**RESULTS:**

the prevalence of satisfaction at work corresponded to 66.4%. Factors directly associated with satisfaction: higher-level function (except physicians and psychologists), work time of six months or less, making a larger number of home visits, good supervision by the team, possibility to make collective choices and take courses.

**CONCLUSIONS:**

the satisfaction is associated with the work organization and conditions and demonstrates the need to invest in team supervisions, in process that democratize the services and in the workers' training.

## Introduction

Mental Health is a global priority for public health in the 21^st^ century. Different actions are needed to consolidate care models that rescue the citizenship of individuals in mental suffering. Therefore, the Brazilian services for care without institutionalization, within the user's territory, structured based on the Psychiatric Reform, increased numerically and reveal peculiarities in the different regions of the Brazilian territory.

The expansion is verified particularly in the network of Psychosocial Care Centers (CAPS), which in 2004 consisted of 689 services and at the end of 2010 reached the sum of 1,620 services implemented in Brazil^(^ 1 ^)^. Among the Brazilian regions, the South presents the best CAPS coverage, with an indicator of 0.87 CAPS/100,000 inhabitants, higher than the Brazilian average of 0.66 CAPS/100,000 inhabitants^(^ 1 ^)^.

The consolidation of the substitutive service network presupposes the problematization and organization of its work processes to consider the exchange of knowledge and practices among the professionals, as well as the valuation of creative potentials and individual competences^(^ 2 ^)^. Deinstitutionalized care comes with challenges that stimulate workers, and can also cause suffering, burden and lack of satisfaction at work.

The repercussions of work derive from the work conditions (physical, mechanical, chemical and biological pressures of the work station) as well as the organization of work (prescribed operation mode, distribution of responsibilities, hierarchy, command modalities and socioprofessional relations, among others)^(^ 3 ^)^. In mental health, daily contact with people in mental suffering is added to these factors, constituting a set that affects the workers' satisfaction and, consequently, their well-being and mental health, whose influence is perceived in the quality of user care and, therefore, of the services^(^ 4 ^)^.

Brazilian quantitative studies adopt concepts that relate satisfaction and dissatisfaction at work as opposite phenomena and use scales to verify them. The use of validated instruments favors the assessment of satisfaction at work in practice^(^ 5 ^)^. Nevertheless, the prevalence of satisfaction at work in Brazilian community mental health services has not been assessed. In the USA, it corresponded to 59% according to the Job Satisfaction Survey, in a sample of 176 technicians^(^ 6 ^)^, and 90% according to the Minnesota Job Satisfaction Questionnaire, in 98 professionals^(^ 7 ^)^.

The factors related to greater satisfaction at work in community mental health services include: greater autonomy^(^ 8 ^-^ 9 ^)^, observation of rapid changes at the service, benefits of teamwork, keeping the clients outside the hospital^(^ 9 ^)^ and organizational support^(^ 10 ^)^. And lesser satisfaction has been associated with administrative tasks and a large number of cases^(^ 11 ^)^, inappropriate physical structure, lack of human and material resources^(^ 12 ^-^ 13 ^)^, devaluation in the workplace^(^ 8 ^)^ and greater burden at work^(^ 8 ^,^ 13 ^-^ 14 ^)^.

To get to know the repercussions of work in community mental health services, the objectives in this study were to analyze the prevalence of satisfaction and identify the associated factors in workers from Psychosocial Care Centers in the three states that constitute the South of Brazil.

## Method

Cross-sectional study and excerpt of the research CAPSUL II^*^, undertaken in 2011 to assess the mental health care offered at the CAPS in the South of Brazil, funded by the Ministry of Health. The data studied here were collected through a questionnaire that was self-applied to the workers of the 40 services drafted for the study, among the 308 existing CAPS, and obtained between August and December 2011. The CAPS sample was structured according to the service supply in the three states within the region, the population concentration per geographic macro-region and the guaranteed presence of the capitals, besides the different CAPS models (I, II, III and excluding CAPSchildren and CAPSalcohol and drugs).

At the 40 CAPS included in the study, all 658 active workers were invited to participate and 546 answered the questionnaire, which permitted estimated a 66% prevalence of satisfaction, with a 4.0 error margin and 95% Confidence Interval. To calculate associations, an alpha coefficient of 5% was used, with 80% statistical power to detect a minimal relative risk of 1.5 in the exposures and a 2:1 index between not-exposed/exposed.

The satisfaction outcome was defined based on the application of the* Assessment Scale of Team Satisfaction in Mental Health Services *(SATIS-BR), which is self-administered and contains 32 quantitative items. Each question shows answers arranged on a five-point Likert scale, in which 1 = very dissatisfied, 2 = dissatisfied, 3 = indifferent, 4 = satisfied and 5 = very satisfied. The SATIS-BR was developed by the World Health Organization (WHO) in a multicenter project and validated in Brazil^(^ 5 ^)^ based on a Canadian study, with a high internal consistency coefficient (α=0.89).

The prevalence of satisfaction was calculated based on the mean global satisfaction scores, stratified in five segments: 1 = 1.0 - 1.5 (very dissatisfied); 2 = 1.51 - 2.5 (dissatisfied); 3 = 2.51 - 3.5 (indifferent); 4 = 3.51 - 4.5 (satisfied); 5 = 4.51 - 5.0 (very satisfied), their proportions were verified^(^ 13 ^,^ 15 ^)^ and groups 4 and 5 were identified as the presence of satisfaction.

The remaining variables were organized according to six hierarchical levels, according to the theoretical determination model^(^ 16 ^)^; in which the level furthest from the outcome consisted of demographic variables (sex, age, marital situation and education) and the type of service (CAPS I, CAPS II, CAPS III) and the second level of work insertion variables (salary, workload at the CAPS, workload at another service, function in CAPS, employment relationship and length of work in CAPS).

The third level included behavioral (smoking and alcohol consumption) and work organization variables (home visit, group care and team meetings).

At the fourth level, the workers' assessment of the supervision were explored, subdivided in: by the secretary of health, by the team and by the community, on a scale from 0 to 10; after collecting these variables, they were categorized as bad (0/3), intermediary (4/6) and good (7/10). This same level includes the characteristics of CAPS work, represented by: lack of tools for work, possibility to make collective choices, possibility to take courses.

The fifth level consisted of variables related to cases of absence in a six-month period and to the health conditions, which were: self-referred health problems and suspicions of minor psychiatric disorders, using the Self Report Questionnaire 20 (SRQ 20). The SRQ 20 consists of 20 questions with a yes-or-no answer, translated and validated to Portuguese^(^ 17 ^)^; to define the prevalence of suspected minor psychiatric disorders, the cut-off point was set as eight or more positive answers for women and six or more positive answers for men.

The level that was closest to the outcome satisfaction at work, the sixth, consisted of the assessment of the work burden, measured using the Assessment Scale of the Impact of Work in Mental Health Services (IMPACTO-BR), developed by WHO and validated in Brazil^(^ 5 ^)^; it presents good item homogeneity and high internal consistency with α = 0.87. IMPACTO-BR contains 18 items, each with answers arranged on a five-point Likert scale, where 1 = not at all, 2 = not much, 3 = more or less, 4 = a lot, 5 = extremely, based on which the global average was calculated and stratified according to the original five-point scale^(^ 12 ^)^.

The data were analyzed in the statistical program STATA 9.0. The bivariate analysis examined the prevalence of satisfaction in each variable studied. The associations were tested using the chi-square test, and differences with p ≤ 0.05 were considered significant. The logistic regression model was applied, the gross and adjusted odds ratios were calculated with a 95% confidence interval (95% CI) and backward selection. Variables with p ≤ 0.10 were maintained in the model.

Approval for the study protocol was obtained from the Ethics Committee at the School of Nursing of *Universidade Federal de Pelotas* (No 176/2011) and the ethical principles were guaranteed according to the Standards and Regulatory Guidelines of Research Involving Human Beings - Resolution CNS 196/96, use of free and informed consent form, guarantee of right to non-participation at any time in the research and anonymity of the interviewee.

## Results

The study participants were 546 workers (83% of the 658 workers at the 40 services), mostly women (79.7%), with a mean age of 37.5±10.8 years, who held a higher education degree (54%) and a mean length of experience at the services of 39.6±45 months. The prevalence level of satisfaction found in the study sample corresponded to 66.4% and the mean global satisfaction was 3.6 (range from 1 to 5).

The following variables showed to be statistically associated with satisfaction at work in the gross analysis: age; education, with a downward trend as education increases; function in CAPS; employment relationship; length of experience in CAPS; supervision by the health secretary, by the team and by the community; lack of tools for work; possibility to make collective choices and take courses; absence from work in previous six months; self-referred health problem; minor psychiatric disorders and presence of work burden (Tables 1 and 2).

**Table 1:** Prevalence of satisfaction according to demographic variables, type of service, insertion in work, behavioral variables and the respective Odds Ratio (OR), 95% confidence intervals (95% CI) and p-values, in CAPS workers from the South of Brazil, 2011. (N=546)

Variable		n	Satisfaction	OR (95% CI)	p value
Sex					0.869
	Male	111	65.8%	1.00	
	Female	435	66.6%	1.03(0.67-1.61)	
Age					0.012
	≤ 25 years	73	67.1%	1.00	
	26 to 35 years	180	62.8%	0.83(0.46-1.47)	
	36 to 45 years	143	62.9%	0.83(0.46-1.51)	
	≥ 46 years	144	74.3%	1.42(0.77-2.62)	
Marital Situation					0.199
	Single	190	66%	1.00	
	Fixed partner	286	68.5%	1.10(0.74-1.63)	
	Separated/divorced/widowed	67	57%	0.66(0.38-1.18)	
Education					0.003^*^
	Primary Education	61	78.7%	1.00	
	Secondary Education	186	71.5%	0.68(0.34-1.35)	
	Undergraduate	126	60%	0.41(0.20-0.83)	
	Graduate	173	61.3%	0.43(0.22-0.85)	
Type of CAPS					0.060
	CAPS I	257	71.7%	1.00	
	CAPS II	181	62.5%	0.66(0.44-0.99)	
	CAPS III	108	61%	0.63(0.39-1.00)	
Salary					0.157
	≤ 2 Brazilian Minimum W.	208	70.2%	1.00	
	>2 to 5 Brazilian Minimum W.	216	62%	0.69(0.46-1.04)	
	> 5 Brazilian Minimum W.	70	61.4%	0.67(0.38-1.19)	
Workload at CAPS					0.125
	1 to 20 Hours/week	166	69.1%	1.00	
	21 to 30 Hours/week	152	59.9%	0.67(0.42-1.06)	
	31 to 60 Hours/week	224	69.2%	1.00(0.65-1.55)	
Workload Other Location					0.102
	0 Hour	382	68.6%	1.00	
	1 to 18 Hours	46	71.7%	1.16(0.59-2.29)	
	20 to 30 Hours	77	59.7%	0.68(0.41-1.12)	
	31 to 135 Hours	41	52.5%	0.59(0.26-0.98)	
Function in CAPS					0.007
	Physician and Psychologist	111	54.1%	1.00	
	Other higher-level functions^ †^	170	67.5%	1.76(1.08-2.88)	
	Secondary and Primary-level Functions	265	74.7%	2.07(1.31-3.28)	
Employment Contract					0.004
	CLT/Statutory	359	61.8%	1.00	
	Temporary contract	159	74.7%	1.82(1.20-2.76)	
Length of experience in CAPS					<0.001
	1 to 6 months	133	83.5%	1.00	
	7 to 24 months	143	59.9%	0.29(0.17-0.52)	
	25 to 250 months	267	61.1%	0.31(0.18-0.52)	
Smoking					0.127
	No	438	67.3%	1.00	
	Yes	59	73.9%	1.31(0.71-2.40)	
	Former Smoker	46	54.4%	0.58(0.31-1.07)	
Alcohol consumption					0.083
	Does not drink	208	72.1%	1.00	
	1 per month or more	138	62.3%	0.64(0.40-1.01)	
	2 per month or more	182	63.2%	0.66(0.43-1.02)	

* Trend Test †Nurse, social worker, pedagogue, occupational therapist, physical educator, plastic artist, musical technician, nutritionist, pharmacist, physiotherapist, speech therapist

**Table 2:** Prevalence of satisfaction according to the variables work organization, assessment of supervision, work organization, health conditions, burden and respective Odds Ratios (OR), 95% confidence intervals (95% CI) and p-values, in CAPS workers from the South of Brazil, 2011. (N=546)

Variable		n	Satisfaction	OR (95% CI)	p value
Home Visits					0.070
	Up to 5 HV/month	356	64.3%	1.00	
	Between 6 and 40 VD/month	87	74.4%	1.61(0.95-2.74)	
Care in Groups					0.227
	0 Care in Groups (CG)	162	71%	1.00	
	1 to 12 CG/month	176	66%	0.78(0.49-1.24)	
	13 to 360 CG/month	105	61%	0.63(0.37-1.07)	
Team Meetings (TM)					0.615
	0 TM	86	66.3%	1.00	
	1 to 4 TM/month	291	63.5%	0.88(0.53-1.46)	
	5 to 31 TM/month	93	68.8%	1.12(0.60-2.10)	
Supervision MHS					<0.001
	Bad (0,1,2,3)	205	55.6%	1.00	
	Intermediary (4,5,6)	96	60.4%	1.21(0.74-1.99)	
	Good (7,8,9,10)	204	78.9%	3.15(2.03-4.89)	
Supervision Team					<0.001
	Bad or Unsatisfactory	67	29.9%	1.00	
	Intermediary	67	58.2%	3.27(1.60-6.68)	
	Good	373	74.2%	6.76(3.81-11.98)	
Supervision Community					<0.001
	Bad or Unsatisfactory	224	54.5%	1.00	
	Intermediary	92	59.8%	1.24(0.75-2.03)	
	Good	182	81.8%	3.74(2.36-5.93)	
Lack of tools					<0.001
	No	249	80.7%	1.00	
	Yes	283	53%	0.27(0.18-0.40)	
Collective choices					<0.001
	No	47	40.4%	1.00	
	Yes	486	68.5%	3.20(1.73-5.90)	
Can take courses					<0.001
	No	137	48.2%	1.00	
	Yes	379	72%	2.76(1.84-4.12)	
Absences in previous 6 months					0.008
	No	335	71%	1.00	
	Yes	204	59%	0.61(0.43-0.87)	
Health Problems					0.037
	No	373	69.1%	1.00	
	Yes	148	59.5%	0.65(0.44-0.98)	
SRQ – 20					0.003
	Negative	381	71.1%	1.00	
	Positive	28	42.9%	0.30(0.14-0.66)	
Burden					<0.001
	No	272	78.9%	1.00	
	Yes	269	54.3%	0.32(0.22-0.47)	

After adjustments, the chances that the CAPS workers were satisfied with their work were 86% higher for higher-level workers (nurse, social worker, pedagogue, occupational therapist, physical educator, plastic artist, music technician, nutritionist, pharmacist, physiotherapist, speech therapist) than for physicians and psychologists; and 84% higher for workers with between six and 40 home visits per month when compared to those with up to five home visits per month. In addition, assessing the supervision by the team as good increased the chances of satisfaction by 2.9 in relation to those workers who assessed it as bad; and the possibility of making collective choices and taking courses increased the chances of being satisfied by 6.4 and 1.3 times, respectively (Table 3).

**Table 3:** Adjusted analysis of effect of independent variables on satisfaction at work at Psychosocial Care Centers in the South of Brazil, 2011. (N=546)

Variable		Adjusted OR* (95% CI)	p value
Education			0.045
	Primary Education	1.00	
	Secondary Education	0.60(0.28-1.28)	
	Undergraduate	0.23(0.08-0.65)	
	Graduate	0.26(0.08-0.82)	
Function in CAPS			0.012
	Physician and Psychologist	1.00	
	Other high-level functions^†^	1.86(1.11-3.12)	
	Secondary and Primary-level Functions	0.70(0.27-1.81)	
Employment Contract			0.052
	CLT/Statutory	1.00	
	Temporary Contracts	1.57(0.99-2.48)	
Length of experience in CAPS			0.0001
	1 to 6 months	1.00	
	7 to 24 months	0.30(0.17-0.55)	
	25 to 250 months	0.36(0.20-0.63)	
Home Visits			0.03
	Up to 5 HV/month	1.00	
	Between 6 and 40 HV/month	1.84(1.04-3.25)	
Supervision Team			0.001
	Bad or Unsatisfactory	1.00	
	Intermediary	3.50(1.42-8.61)	
	Good or Satisfactory	3.94(1.86-8.38)	
Lack of tools			0.0002
	No	1.00	
	Yes	0.38(0.23-0.64)	
Collective Choices			0.001
	No	1.00	
	Yes	7.39(1.86-29.31)	
Can take Courses			0.006
	No	1.00	
	Yes	2.27(1.27-4.06)	
SRQ 20			0.09
	Negative	1.00	
	Positive	0.34(0.12-0.94)	
Burden			0.022
	No	1.00	
	Yes	0.48(0.25-0.90)	

* According to hierarchical model †Nurse, social worker, pedagogue, occupational therapist, physical educator, plastic artist, musical technician, nutritionist, pharmacist, physiotherapist, speech therapist

The variables that showed to be inversely associated with satisfaction after adjustments were: working at CAPS between seven and 24 months, which reduced the chances of being satisfied by 70% when compared to workers with six months or less of experience at the services; lack of tools for work, which dropped the chances of satisfaction by 62%; and work burden, which reduced the chances of being satisfied at work in Psychosocial Care Centers by 52% (Table 3).

The variables training, employment contract and minor psychiatric disorders were maintained in the model, although they did not demonstrate significance, as they are potentially confounding.

## Discussion

In line with the literature, this study identified a strong association between satisfaction and work conditions and with the organization of work in the CAPS. The workers' individual characteristics were less significant among the factors associated with satisfaction.

The mean global satisfaction coefficient identified, corresponding to 3.6, is close to other Brazilian studies. These assessed satisfaction in workers active in services that were implemented after the changes resulting from the Brazilian Psychiatric Reform and identified global satisfaction scores of 3.43 and 3.59 using SATIS-BR^(^ 13 ^-^ 14 ^)^, classified as bordering on indifference, on a scale from 1 to 5 points. Other studies identified higher mean satisfaction rates (4.05 and 4.02), but their samples characterize a single service^(^ 15 ^,^ 18 ^)^. Intermediary satisfaction scores were identified in studies in Italy, which used a non-validated questionnaire^(^ 19 ^)^ and the Minnesota Satisfaction Questionnaire^(^ 20 ^)^.

Nevertheless, the Brazilian studies did not assess the prevalence of satisfaction at work in community mental health services, in this study identified as 66.4%. In the USA, the prevalence rate corresponded to 59% when using the Job Satisfaction Survey, in a sample of 176 technicians^(^ 6 ^)^, and to 90% when using the Minnesota Job Satisfaction Questionnaire, in 98 professionals^(^ 7 ^)^.

The short insertion period in deinstitutionalized services for people in mental services permits less exposure to the daily reality at the CAPS. Consequently, the workers may not have experienced a range of situations to permit an assessment, like workers with longer experience at CAPS. These evidences support the findings that associated five to ten years of work experience in Mental Health with lower satisfaction levels^(^ 13 ^)^; and differ from a study in the United Kingdom that associated five or more years of work with higher satisfaction levels^(^ 11 ^)^.

When the CAPS team uses the home visit strategy, it mainly intends to enable the family to use its own resources, include it in the treatment process and increase the possibilities of bonding with the professionals^(^ 21 ^)^. The workers identified with the enhanced view of the madness phenomenon and with deinstitutionalized and territorial care aim to reintegrate the users with their family and community and, thus, do home visits, is one of the frequent work strategies.

Many professionals get unique interdisciplinary work experience in the CAPS. Higher-education professions in mental health, with different work processes, can articulate them and organize them in a specific manner^(^ 2 ^)^. The results support the identification of the association between satisfaction and autonomy in community health service work in the United Kingdom and the USA^(^ 8 ^-^ 9 ^,^ 11 ^)^ but, nevertheless, did not identify an association with profession/function^(^ 11 ^)^ and a difference in satisfaction between physicians and nurses^(^ 19 ^)^.

The results indicate that greater importance is attributed to the relations established in the work team, through supervision and collective choices, as observed in a study of 209 workers at CAPS and Home-Based Therapeutic Services, in which team difficulties or problems were identified as the most prevalent situation that bothered the workers among the situations mentioned^(^ 22 ^)^.

Besides the situations defined in the teams' internal arrangements, factors associated with satisfaction can be established based on service management, such as the possibility to make collective choices, the possibility to take courses, lack of tools for work. These results are in accordance with findings in the literature that associated satisfaction with organizational support^(^ 10 ^)^, rapid changes in the services^(9) ^and physical structure, human and material resources^(^ 12 ^-^ 13 ^)^.

The work burden was inversely associated with satisfaction, as identified in other studies^(^ 8 ^,^ 11 ^,^ 13 ^-^ 14 ^,^ 20 ^)^. The burden may be related to service management, in the form of an insufficient number of workers to provide user care within the territory, as well as to work organization difficulties. Daily work in CAPS presupposes commitment to psychosocial care and creativity in the search to reinsert the user but, besides a conquest, these premises may represent an obligation and even a burden for the service workers^(^ 23 ^)^.

Satisfaction may be overestimated due to the possible dissatisfaction among the workers who chose not to manifest their opinion and the workers absent from their workplace; also, the use of logistic regression may have expanded the confidence intervals of the associations, increasing the variance of the estimates. In addition, the limitations inherent in a cross-sectional study are considered, which does not identify the changes over time in the variables involved. Possible interferences include the different lengths of the services' existence, as some had a recent history and may not have established routines and defined their functioning yet.

## Final Considerations

The results show a strong association between satisfaction and factors related to the organization and work conditions in the CAPS, based on which reflections are possible and collective actions can be proposed to enhance the workers' satisfaction. Adapting the services' material conditions (physical area, equipment, drugs, material) to comprehensive care and to the range of activities needed for deinstitutionalized care delivery is highlighted.

It is also important to expand and qualify the supervision by the team, including opportunities for discussion and planning in daily service work, as well as decentralized and democratic management processes. Another aspect that should be considered is the workers' training, articulated with the conceptual frameworks and training areas in the CAPS, stimulated and promoted by the managers.

The transition between models is ongoing. The deinstitutionalized care model is not hegemonic and coexists with the asylum model. Workers who believe in the Psychiatric Reform and value the organization forms of their work are the main occupants of the work places in mental health. Enhancing their satisfaction, keeping in mind that one third of the interviewees are dissatisfied at their work, may contribute to the consolidation of the model.
